# Experience-based ambivalence toward medical opioid use among Japanese adults: a nationwide cross-sectional survey using the barriers questionnaire II

**DOI:** 10.1186/s40780-026-00540-y

**Published:** 2026-01-14

**Authors:** Tomofumi Watanabe, Atsunobu Sagara, Tomoya Abe, Hiroshi Kudoh, Satoshi Yoshikawa, Atsushi Ishimura, Hiroyuki Terakado

**Affiliations:** 1 Department of Clinical Epidemiology, School of Pharmacy and Pharmaceutical Sciences, Hoshi University, 2-4-41 Ebara, Shinagawa-ku, Tokyo, Japan; 2 Department of Pharmacy, Japanese Red Cross Medical Center, 4-1-22 Hiroo, Shibuya-ku, Tokyo, Japan; 3 Department of Pharmacy, Saitama Cancer Center, 780 Komuro, Ina- machi, Kitaadachi-gun, Saitama, Japan; 4 Department of Pharmacy, Japan Institute for Health Security, National Center for Global Health and Medicine, 1-21-1 Toyama, Shinjuku-ku, Tokyo, Japan; 5 Faculty of Pharmacy, Iryo Sosei University, 5-5-1 Chuo-dai Iino, Iwaki, Fukushima Japan; 6 Department of Clinical Pharmacy Assessment, School of Pharmacy and Pharmaceutical Sciences, Hoshi University, 2-4-41 Ebara, Shinagawa-ku, Tokyo, Japan

**Keywords:** Medical opioids, Cancer pain management, The Japanese version of the barriers questionnaire II (JBQ-II), Ambivalence, Family experience, Willingness to use opioids, Public attitudes

## Abstract

**Background:**

Pain is one of the most distressing symptoms in patients with cancer. Although established guidelines now recommend appropriate opioid use, even for mild-to-moderate pain, Japan’s prescription volume remains markedly lower than that of Western countries. Public ambivalence, recognizing opioids as effective yet fearing dependence and adverse effects, may contribute to this underuse. However, little is known about how indirect exposure to opioids through family members’ cancer treatments shapes such attitudes. This study examined the perceived barriers to and willingness to use medical opioids among cancer-free adults, focusing on the influence of family experiences with opioid use.

**Methods:**

A cross-sectional web-based survey was conducted on July 30, 2025, among 618 Japanese adults aged 20–49 years without a history of cancer. Participants were categorized into three groups: Opioid+ (family with cancer and opioid use), Opioid− (family with cancer but no opioid use), and None (no family history of cancer). Psychological barriers were assessed using the Japanese version of the Barriers Questionnaire II (JBQ-II), comprising an overall score and five subscales. Willingness to use opioids in a hypothetical mild-to-moderate cancer pain scenario was rated on a 10-point Likert scale. Group differences were analyzed using Kruskal–Wallis and Steel–Dwass tests (two-tailed *p* < 0.05).

**Results:**

Compared with the None group, the Opioid + group showed significantly higher scores for overall JBQ-II (3.23 vs. 3.11, *p* < 0.01), Physiological Effects (3.47 vs. 3.32, *p* < 0.05), Harmful Effects (3.32 vs. 3.12, *p* < 0.001), and Disease Progression (3.65 vs. 3.39, *p* < 0.01), but lower Fatalism scores (2.25 vs. 2.46, *p* < 0.05). Willingness to use medical opioids was also higher in the Opioid + group (7.43) than in the Opioid− (6.55, *p* < 0.01) and None groups (6.59, *p* < 0.01).

**Conclusions:**

Indirect exposure to opioids through family members’ cancer treatment was associated with ambivalent attitudes, characterized by greater recognition of barriers alongside increased willingness to use medical opioids. Addressing this experience-based ambivalence through evidence-based education, individualized counseling using the JBQ-II, and family-involved communication support may help promote the safe and appropriate use of medical opioids in Japan’s cultural and public health contexts.

**Clinical trial number:**

Not applicable.

## Background

Pain is among the most distressing symptoms experienced by patients with cancer. Reports indicate that 55% of the patients undergoing anticancer drug therapy and 66% of those with advanced, metastatic, or terminal diseases experience cancer-related pain [1, 2]. Traditionally, opioids have served as the mainstay of pharmacological treatment for moderate-to-severe cancer pain [3–5]. However, in the 2018 revision of the guidelines for cancer pain management, the World Health Organization (WHO) expanded the indications for opioid use to include mild-to-moderate pain [6, 7]. In Japan, the appropriate use of medical opioids has been promoted following the enactment of the Basic Act on Cancer Control in 2007 [8].

Despite these efforts, the domestic prescription volume of medical opioids in Japan remains low, approximately one-eighth of that in Western countries, according to international standards [9]. Furthermore, according to the Cabinet Office’s “Public Opinion Survey on Cancer Control (2023),” a growing understanding that “proper use is effective and safe” coexists with perceptions of it as a “last resort” and concerns about adverse events and dependence, revealing an ambivalent attitude among the general public [10, 11]. Such ambivalence may influence the acceptance of medical opioids in clinical practice.

The Japanese version of the Barriers Questionnaire II (JBQ-II) was developed to assess psychological barriers to cancer pain management, and its reliability and validity have been established [12]. The JBQ-II comprises five subscales—Physiological Effects, Communication, Harmful Effects, Disease Progression, and Fatalism—allowing for a multidimensional evaluation of perceptions related to medical opioid use. However, studies applying the JBQ-II to members of the general public without a history of cancer are limited, and the influence of family experiences with medical opioid use during cancer treatment on barrier perception and acceptability has not been fully elucidated [12, 13].

This study aimed to compare the perceptions of barriers to and intentions toward medical opioid use, as measured by the JBQ-II, among cancer-free members of the general public. Particular attention was given to individuals in their 20s to 40s, who are more likely to face future cancer diagnoses or family caregiving roles. By examining differences based on the presence or absence of family experience with medical opioid use, this study seeks to clarify the characteristics of experience-based attitude formation and provide foundational insights for educational and public awareness initiatives promoting the appropriate use of medical opioids.

## Methods

### Survey period and participants

A cross-sectional web-based questionnaire survey was conducted on July 30, 2025. Recruitment was performed using the online research panel of Macromill Inc. (Tokyo, Japan). To prevent fraudulent responses, Macromill implemented strict quality control measures, including trap question surveys conducted every 6 months and mandatory annual updates of monitor registration information [14, 15]. As of July 2025, 30,398 individuals in the panel met the initial screening criteria.

#### Eligibility criteria

The specific inclusion and exclusion criteria were as follows:

- Inclusion criteria: Men and women aged 20–49 years residing in Japan who had no self-reported personal history of cancer.
- Exclusion criteria: Healthcare professionals (e.g., physicians, nurses, and pharmacists) were excluded to avoid professional bias.

#### Group definitions and sampling strategy

Respondents were categorized into three groups based on their family history of cancer and experience with medical opioid use:

- None: No family history of cancer.
- Opioid−: Family history of cancer without medical opioid use experience.
- Opioid+: Family history of cancer with medical opioid use experience.

To ensure sufficient statistical power for intergroup comparisons, we set a target sample size of approximately 600 participants across the three groups (about 200 per group). We employed a quota sampling method to ensure comparability between the groups, and recruitment was automatically terminated when the number of valid responses reached 206 in each group. Consequently, a total of 618 participants (206 per group) were included in the final analysis.

### Survey items

Before the JBQ-II questions, the following instruction was displayed to define the terminology and context: “This survey concerns ‘cancer pain control’ and the ‘medical opioids (analgesics)’ used for this purpose. The terms ‘painkillers’ and ‘medicines’ used in the questions all refer to ‘medical opioids’.”

This ensured that participants distinguished medical opioids from general analgesics without receiving extensive education that could bias their responses.

#### Barriers to cancer pain management

Barriers were assessed using the JBQ-II [12]. Due to copyright restrictions, the specific items are not listed in this article; permission from the original developers is required to access or use the scale. The scale comprises five subscales: Physiological Effects (11 items), Communication (7 items), Harmful Effects (6 items), Disease Progression (3 items), and Fatalism (3 items). Each item is rated on a six-point Likert scale (0 = strongly disagree to 5 = strongly agree). The Fatalism subscale (three items) was reverse-scored. The overall and subscale scores were calculated as the mean of the corresponding items, with higher scores indicating greater perceived barriers.

#### Intent to use medical opioids in a hypothetical situation

Participants were presented with the following scenario and asked to rate their intention to use medical opioids on a 10-point Likert scale (1 = I would prefer not to use medical opioids, if possible, to 10 = I would like to use medical opioids to relieve pain). Higher scores indicated a stronger intent to use.

Assume you are experiencing cancer-related pain that interferes with your daily life, but the pain level is tolerable. In this situation, if your physician recommends the use of medical opioids to relieve pain, would you want to use them?

### Statistical analysis

Intergroup comparisons of categorical variables (sex and residential area) were conducted using the chi-squared (χ²) test. When significant differences were detected, multiple comparisons were performed using Bonferroni correction. For continuous variables (age, JBQ-II subscale scores, and intent to use medical opioids), the Kruskal–Wallis test was applied, followed by the Steel–Dwass method for post-hoc pairwise comparisons.

All statistical analyses were performed using JMP Pro 18 software (SAS Institute Inc., Cary, NC, USA). Microsoft Excel (Microsoft Corp., Redmond, WA, USA) was used as an auxiliary tool for the Bonferroni correction. The level of statistical significance was set at *p* < 0.05 (two-tailed).

## Results

### Participant characteristics

A total of 618 valid responses were obtained, with 206 participants in each group (Opioid+, Opioid−, and None). No significant differences were observed in age distribution, sex, or residential area between the groups. Therefore, the influence of demographic characteristics was considered limited, and subsequent analyses were conducted based on comparisons across the three groups (Table 1).

**Table 1:** Participant characteristics

	None (*n* = 206)	Opioid- (*n* = 206)	Opioid+ (*n* = 206)	Total (*N* = 618)	*p* value
Age					
Means ± SD	41.5 ± 5.8	42.3 ± 5.2	42.2 ± 5.3	42.0 ± 5.4	0.412
Sex					
Male/Female	92/114	90/116	97/109	279/339	0.775
Area					
Hokkaido	4	8	12	24	0.467
Tohoku	17	15	14	46	
Kanto	70	65	74	209	
Chubu	34	41	30	105	
Kinki	44	39	42	125	
Chugoku	18	8	12	38	
Shikoku	5	7	5	17	
Kyushu	14	23	17	54	

Comparisons of participant characteristics were performed using the chi-squared (χ²) test for categorical variables (Sex and Area) and the Kruskal–Wallis test for continuous variables (Age). A significance level of *p* < 0.05 was considered statistically significant

SD Standard Deviation

### JBQ-II scores by family history group

**Fig. 1:**
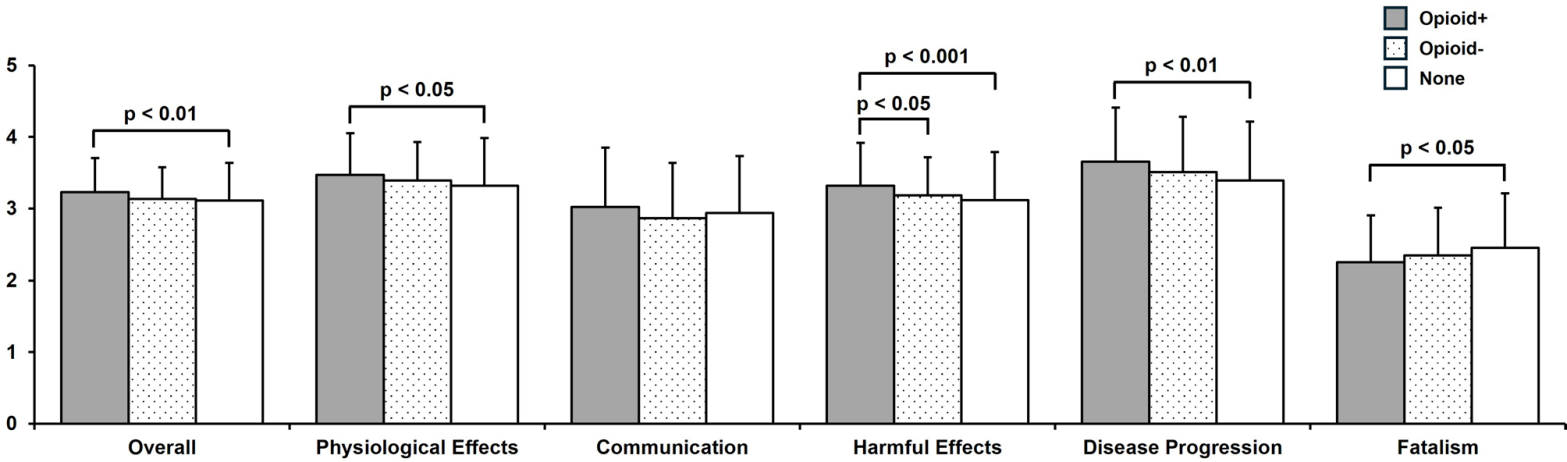
Mean scores (± SD) for each JBQ-II overall and subscales are presented. The Kruskal–Wallis test was followed by the Steel–Dwass post hoc test. *p* < 0.05 was considered statistically significant

Figure 1 presents a comparison of JBQ-II overall and subscale scores across the three groups. Significant intergroup differences were found in four domains: Physiological, Harmful, Disease Progression, and Fatalism.

The Opioid + group had significantly higher scores than the None group in terms of Overall (3.23 ± 0.48 vs. 3.11 ± 0.53, *p* < 0.01), Physiological Effects (3.47 ± 0.58 vs. 3.32 ± 0.67, *p* < 0.05), and Disease Progression (3.65 ± 0.76 vs. 3.39 ± 0.83, *p* < 0.01). In Harmful Effects, the Opioid + group (3.32 ± 0.60) scored significantly higher than both the Opioid − group (3.18 ± 0.53, *p* < 0.05) and the None group (3.12 ± 0.67, *p* < 0.001). Conversely, the Fatalism score was significantly lower in the Opioid + group (2.25 ± 0.65) than in the None group (2.46 ± 0.75, *p* < 0.05). No significant group differences were observed in Communication (*p* = 0.062).

### Willingness to use medical opioids in a hypothetical scenario

**Fig. 2:**
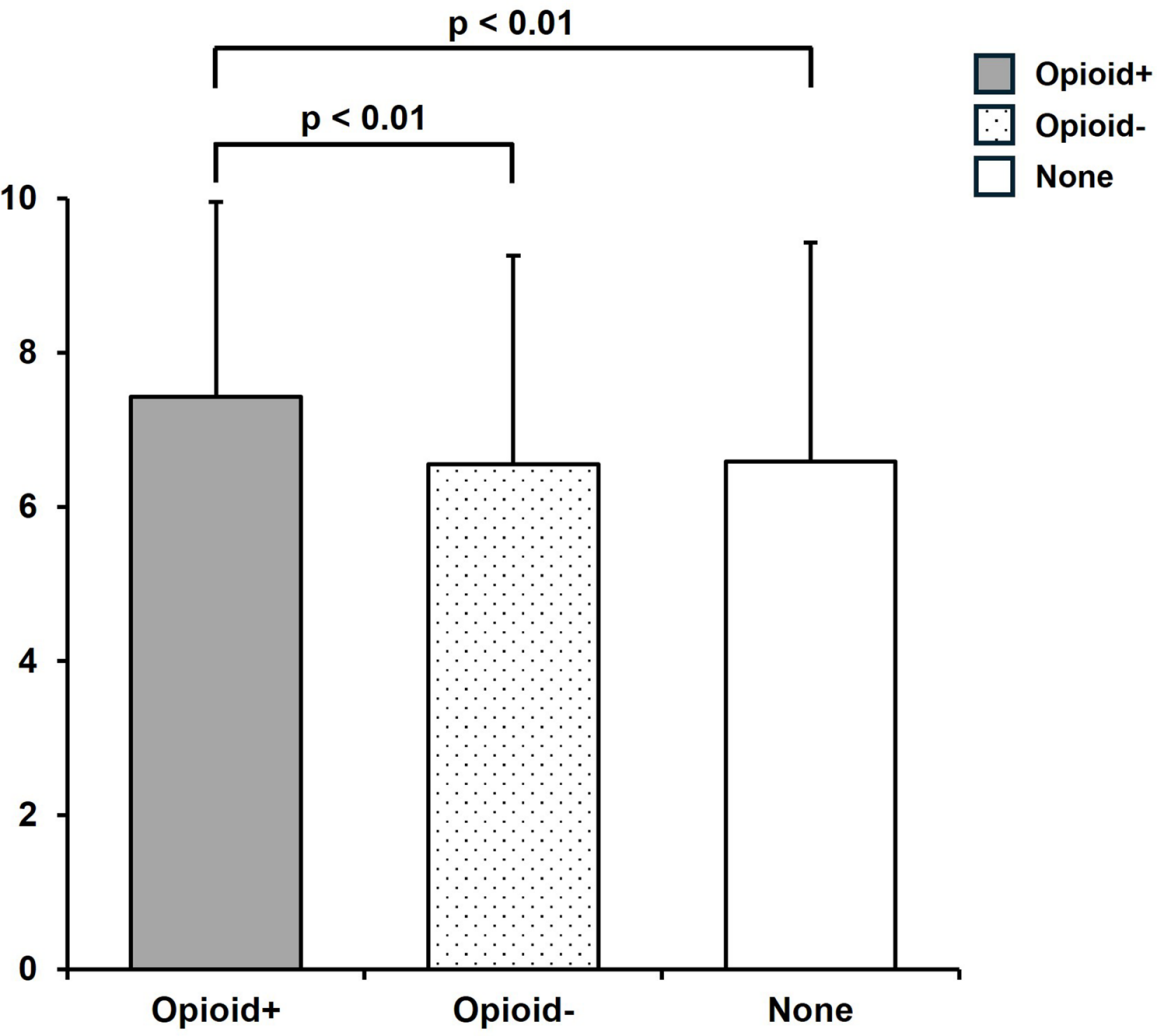
Mean scores (± SD) of willingness to use medical opioids in a hypothetical mild-to-moderate cancer pain scenario are presented. The Kruskal–Wallis test was followed by the Steel–Dwass post hoc test. *p* < 0.05 was considered statistically significant

Figure 2 shows a comparison of the willingness to use medical opioids in a hypothetical scenario among the three groups. The willingness to use medical opioids for mild-to-moderate cancer pain was highest in the Opioid + group (7.43 ± 2.53) and was significantly higher than that in the Opioid- group (6.55 ± 2.71, *p* < 0.01) and the None group (6.59 ± 2.85, *p* < 0.01). No significant difference was observed between the Opioid- group and the None group (*p* = 0.992).

## Discussion

### Statement of key findings

This is the first study in Japan to examine the impact of indirect exposure to medical opioids through a family member’s cancer treatment on psychological barriers and the willingness to use them through a nationwide cross-sectional survey of the general public without a personal history of cancer. Individuals in their 20s to 40s represent the generation most likely to face a future cancer diagnosis or assume caregiving responsibilities for family members. Therefore, understanding attitude formation in this demographic is crucial for promoting the appropriate use of medical opioids within society. The findings indicate that family experience with medical opioid use increased concerns about adverse events and disease progression while simultaneously promoting acceptance of medical opioids and potentially contributing to the promotion of appropriate use.

### Interpretation

Family experience appeared to exert an ambivalent influence on attitude formation toward medical opioids. Specifically, witnessing a family member’s opioid use may heighten concerns about adverse effects (e.g., constipation, somnolence, and nausea) and disease progression through direct observation of physical changes, thereby increasing the perceived barriers related to Harmful Effects, Physiological Effects, and Disease Progression [16–18]. Conversely, observing effective pain relief may reduce fatalistic beliefs and enhance recognition of the efficacy and acceptability of medical opioids. As a result, an ambivalent attitude emerges—simultaneously characterized by “concern-driven hesitation” and “high acceptability.” This ambivalence mirrors the prevailing perception of medical opioids in Japanese society, where viewing them as a “last resort” and anxiety regarding adverse effects or dependence coexist with a growing understanding that they are “effective and safe when used appropriately” [10, 11].

A previous study validating the Japanese version of the JBQ-II (Sakakibara et al. [12]) reported that healthcare professionals demonstrated extremely low barrier scores (mean item score ≈ 0.74), while patients with cancer pain showed moderate scores (mean ≈ 1.97). In contrast, our study revealed substantially higher barrier scores among the general population. This comparison suggests that while the impact of having a family member with opioid experience is significant, it is relatively modest compared to the impact of professional training or personal patient experience. Nevertheless, the consistent differences observed in our study indicate that family experience exerts a definite influence.

Importantly, despite these persistent barriers, family experience with medical opioids increased willingness to use them for mild-to-moderate cancer pain. Current cancer pain management guidelines recommend the appropriate use of opioids, even for mild to moderate pain, and the higher willingness to use observed in the Opioid + group aligns with this clinical direction [6, 7]. The observed increase in willingness scores, however, was smaller than our initial anticipation of approximately 2 to 3 points. This discrepancy likely underscores the complexity of the family experience. While the recognition of efficacy acts as a strong driver for willingness, the heightened concerns regarding adverse effects (as indicated by the barrier scores) likely acted as a counterbalancing force, dampening the overall increase. Despite this, the shift holds practical implications. This pattern may reflect a form of benefit-oriented ambivalence, wherein expectations regarding the necessity and efficacy of pain relief outweigh concerns regarding potential adverse effects. Moreover, the absence of differences in willingness between the Opioid- and the None groups suggests that direct exposure to opioids, rather than a family history of cancer, is the primary determinant of attitude formation. In other words, among the general public, first-hand successful experiences and family narratives regarding opioids appear to exert a stronger influence on attitude formation than abstract or theoretical knowledge.

### Clinical and public health implications

The findings underscore the importance of educational and supportive strategies to alleviate concerns and enhance acceptance through experience-based understanding. Specifically, four practical approaches are proposed.

1. Evidence-based myth corrections

It is essential to disseminate scientifically grounded information on common adverse effects (e.g., constipation and somnolence), low risk of dependence, and the fact that initiating medical opioids does not indicate disease progression or poor prognosis.

(2) Early initiation of opioids

Reframe perceptions that opioids are a “last resort” and emphasize that early initiation for mild-to-moderate cancer pain is a clinically appropriate, evidence-based treatment option.

(3) Individualized counseling using the JBQ-II

Identification of predominant barrier domains through pre-visit screening. For patients with high Disease Progression concerns, emphasize that prescriptions aim at “symptom relief and maintenance of functional capacity,” rather than indicating “disease worsening.” Conversely, for those with high Fatalism scores, reduce anxiety and foster acceptance by communicating that “pain is treatable” and that “accurate pain reporting for dose adjustment is part of the patient’s role.”

(4) Family-involved communication support

Since family experiences influence attitude formation, it is important to involve family members in patient education and decision-making support, with the patient’s consent. This approach helps prevent avoidance or delay of medical care and reduces the risk of delayed pain management due to “gatekeeping.”

### Limitations and future directions

This study had several limitations. First, its cross-sectional design and reliance on a single hypothetical scenario means that self-reported willingness to use opioids may not directly reflect actual behavior in clinical practice. Second, although the use of an online panel may limit generalizability, efforts were made to enhance representativeness through pre-allocation and target sampling. Third, restricting participants to those aged 20–49 years allowed us to focus on the period of social attitude formation and target a population in which educational and awareness interventions were expected to be most effective. However, this design does not capture the perspectives of older adults, among whom cancer incidence is higher, or patients in clinical settings. Fourth, the mediating relationships between the JBQ-II domains and the willingness to use opioids were not examined; therefore, the specific barrier that on being modified resulted in increased acceptability remained unclear. Fifth, detailed participant characteristics, specifically the educational background and the degree and duration of family support for cancer pain, were not investigated. Although educational levels and the intensity of caregiving experiences may distinctly influence attitude formation, this study could not account for these factors.

Future research should clarify the causal relationship between barrier structures and acceptance through prospective mediation analyses that also account for educational background and the quality and quantity of caregiving experiences. Furthermore, longitudinal and repeated cross-sectional studies are warranted to examine how social attitudes toward cancer pain management are formed and evolve over time and to empirically evaluate the modifiability of psychological barriers through educational interventions. This study is expected to provide valuable insights for the development of effective awareness and support programs tailored to different life stages.

## Conclusions

Family members’ experiences with opioid use during cancer treatment were found to shape an ambivalent attitude, characterized by heightened recognition of barriers to cancer pain management and increased acceptance of medical opioids. Promoting guideline-based early initiation, providing individualized support using the JBQ-II, and facilitating family-involved communication may serve as practical strategies for enhancing the appropriate use and safety of medical opioids. These findings offer foundational evidence for the development of culturally tailored public health education and awareness interventions in Japan.

## Data Availability

The datasets used and/or analysed during the current study are available from the corresponding author on reasonable request.

## Abbreviations

JBQ-II: Japanese version of the Barriers Questionnaire II
WHO: The World Health Organization
